# A Novel Proposal for a Bladeless Wind Turbine: Bio-Inspired Design of a Columnar-Cactus Type Mast

**DOI:** 10.3390/biomimetics10100692

**Published:** 2025-10-14

**Authors:** Isaac Hernández-Arriaga, Joaquín Pérez-Meneses, Guillermo Eduardo Mejía-Hernández, Juventino López-Barroso, Cynthia Graciela Flores-Hernández, Daniel Hernández-Arriaga

**Affiliations:** 1 Departamento de Metal Mecánica, Tecnológico Nacional de México, Instituto Tecnológico de Querétaro, Av. Tecnológico s/n Esq. Gral. Mariano Escobedo, Col. Centro Histórico, Querétaro 76000, Mexico; joaquin.pm@queretaro.tecnm.mx (J.P.-M.); guillermo.mh@queretaro.tecnm.mx (G.E.M.-H.); juventino.lb@queretaro.tecnm.mx (J.L.-B.); 2 Departamento de Ciencias Básicas, Tecnológico Nacional de México, Instituto Tecnológico de Querétaro, Av. Tecnológico s/n Esq. Gral. Mariano Escobedo, Col. Centro Histórico, Querétaro 76000, Mexico; cynthia.fh@queretaro.tecnm.mx; 3 Departamento de Ingeniería Eléctrica-Electrónica, Tecnológico Nacional de México, Instituto Tecnológico de Querétaro, Av. Tecnológico s/n Esq. Gral. Mariano Escobedo, Col. Centro Histórico, Querétaro 76000, Mexico; daniel.ha@queretaro.tecnm.mx

**Keywords:** wind energy, bladeless wind turbine (BWT), bio-inspired design, columnar-cactus type mast

## Abstract

This research presents an experimental study on a scaled prototype of a bladeless wind turbine that operates based on the principle of vortex-induced vibrations (VIV-BWT) with the implementation of bio-inspired design of a columnar-cactus type mast. The aerodynamic performance of columnar-cactus type masts with different numbers of ribs was investigated and compared with that of a conventional cylindrical mast. The objective of this novel proposal is to maximize wind energy conversion efficiency through vortex-induced vibrations, thereby enhancing energy generation. The present study focuses on the geometry of the columnar-cactus type mast as a vortex generator, which significantly influences the performance of this type of VIV wind energy harvester. The findings reveal that the geometric configuration of the cactus-inspired mast and the mast angle promote vortex formation, leading to higher lift coefficients and forces. Consequently, this results in greater vortex-induced vibration magnitudes. For instance, at a wind speed of 6.0 m/s and a mast angle of 0°, the 6-rib cactus-type mast exhibits 12 times greater VIV amplitude compared to the conventional cylindrical mast, while the 5-rib and 7-rib cactus-type masts show 2.4- and 2.2-times greater amplitudes, respectively. However, for wind speeds below 5 m/s, the cylindrical mast demonstrates superior VIV performance.

## 1. Introduction

A pressing issue confronting humanity at present is the global accumulation of greenhouse gases (GHGs) resulting from the combustion of carbon-based compounds to satisfy energy demands, particularly in the domains of electricity generation and transportation. In response to this situation, technological advancements have been developed to generate electricity from renewable energy sources. The primary drivers of this development are solar photovoltaic and large-scale wind energy systems [1]. For instance, in 2023, these two sources accounted for 13% of global electricity demand. By the end of 2024, both sources had surpassed hydroelectric power. By 2028, renewable energies will account for more than 42% of global electricity generation. A notable benefit of solar energy systems is their lack of moving components, which contributes to their cost-effectiveness. However, the functionality of these systems is constrained by the availability of daylight, with a maximum energy density of 200 W/m^2^, resulting in an efficiency of approximately 20% [2]. In the context of large-scale wind energy, substantial financial investment is necessary for the construction, transportation, and installation processes, as well as for the acquisition of extensive land areas. As indicated by the third source, wind turbines have been demonstrated to generate noise and thereby have an impact on birds during migration [3]. Furthermore, conventional turbines possess rotating components, including the rotor, gearbox, and bearings, which results in elevated operating and maintenance expenses. In addition, the nominal wind speeds required for optimal operation exceed 6 m per second [4]. Given these considerations, the integration of technology that leverages renewable energy sources has the potential to expedite the transition to more environmentally sustainable technologies and contribute to the reduction in greenhouse gases.

Bladeless wind turbines represent a revolutionary technological advancement that effectively addresses the disadvantages. These turbines operate on the principle of vortex-induced vibrations (VIV), a sophisticated engineering solution that sets them apart in the industry. The following are some of the most important characteristics of these turbines [5,6]: (1) The turbines have no rotating parts, which significantly reduces machine failures and increases their reliability and service life. (2) These turbines operate at low wind speeds, ranging from 2 to 10 m per second, making them suitable for small-scale applications and self-consumption in urban environments. (3) Their cost is approximately 50% of that of conventional horizontal and vertical axis wind turbines. (4) They require minimal space for installation. (5) These products are not noisy, have low environmental impact, and do not compromise bird life through collisions.

On the other hand, the integration of nature-inspired solutions and wind energy has emerged as a promising avenue for enhancing the efficiency and sustainability of wind energy systems [7]. Biomimicry is a rapidly growing field of research that draws inspiration from natural models, systems, and elements to provide innovative design solutions to various problems [8,9], enabling the development of machines that mimic the performance of organisms, especially when their performance exceeds current mechanical technology or offers innovative solutions to existing challenges [10]. Regarding the development of wind turbine systems, biomimicry studies various natural inspirations applicable in different areas, mainly in the aerodynamic and structural design of blades, as this component is responsible for transforming the kinetic energy of wind into mechanical energy. The predominant natural sources that have inspired advances in this field are [7] plants, insects, fish, and birds.

Plant-inspired wind turbine designs have been inspired by the swaying of palm leaves and the aerodynamic efficiency of leaves [11,12,13,14,15], Nile lotus flower [16], maple seeds [17,18,19,20], Dryobalanops aromatica seeds [21], Borneo Camphor seeds [22,23], Petrea Volubilis seeds [24], and a hybridization of the maple seed and Epilobium hirsutum [25]. All the research shows that bio-inspired wind turbines perform better than conventional turbines. For example, they have a higher lift coefficient, a better tip speed ratio (TSR), reduce noise, and operate at lower wind speeds. Other relevant nature-inspired research on wind turbine components includes the following: (1) The implementation of vortex-generating devices inspired by the configuration of peregrine falcon tail feathers, which promote controlled turbulence to delay boundary layer separation on the blades of horizontal-axis wind turbines [26]. Simulations showed a significant reduction in turbulent kinetic energy contours in the wake of the modified turbine compared to the reference configuration. (2) The implementation of the vibrissae of harbor seals for the design of horizontal-axis wind turbine towers [27]. The distinctive three-dimensional structure of vibrissae has a wavy configuration that effectively reduces aerodynamic drag and suppresses vortex formation in the tower wake. In this study, the bionic case showed lower turbulence intensity in its wake compared to that observed in the circular cylinder case and developed a higher power coefficient, this meaning that as power output increases, the bionic structure induces a greater velocity deficit and higher turbulence intensity behind the rotor. (3) The implementation of bio-inspired adhesives that produce more extra-durable and sustainable wind turbine blades [28]. The concept presented in this work includes the development of bio-inspired dual-mechanism-based interface adhesives (e.g., nacre, shells, etc.) that combine mechanical interlocking of fiber and chemical adhesion, which ensures, on the one side, extra-strong attachment during operation and on the other side, possible adhesive joint separation for re-use of the blade parts. Although biomimetic research has focused on conventional horizontal- and vertical-axis wind turbines, no bio-inspired designs have been developed for bladeless wind turbines. This represents an important opportunity. This research is relevant because it improves the aerodynamic performance of turbines through bio-inspired designs that combine the advantages of bladeless turbines with those of conventional turbines, achieving more efficient and sustainable designs.

This research involves an experimental study of a scaled prototype of a bladeless wind turbine with a bio-inspired design of columnar-cactus type mast. The bladeless wind turbine mast was chosen because it captures wind energy and significantly improves aerodynamic performance. The geometry of columnar cactus, inspired by nature, was selected because one of the main functions of cactus ribs is temperature regulation, this is achieved by creating turbulent air currents. These turbulent air currents promote vortex-induced vibrations in bladeless wind turbines, resulting in increased power output. The following hypothesis was developed: “The cross-sectional geometry of a columnar-cactus mast type improves vortex formation when fluid flows over its surface, thereby increasing the aerodynamic performance of a bladeless wind turbine”. However, it is important to note that the columnar cactuses are known for their substantial mass density, which significantly reduces their susceptibility to oscillations when subjected to wind. When the focus is on the cooling function of the ribs, it becomes evident that these structures are designed to optimize geometries for vortex generation, thereby facilitating more efficient convective heat transfer. Therefore, it is logical to consider the transverse geometry of the cactus and treat them as a lightweight structure for wind energy extraction. The study was conducted using three columnar-cactus type masts with 5, 6, and 7 ribs, which are typical in central Mexico. The findings were compared against a conventional cylindrical mast and demonstrated substantial advancements in aerodynamic performance, as evidenced by enhanced coefficients and lift forces. Consequently, these improvements resulted in greater vortex-induced vibrations, signifying an enhanced capacity for wind energy capture. The proposed method of implementing columnar-cactus geometries can be combined with other methods to further maximize turbine performance. For example, it can be combined with the tuning system method.

**Figure 1:**
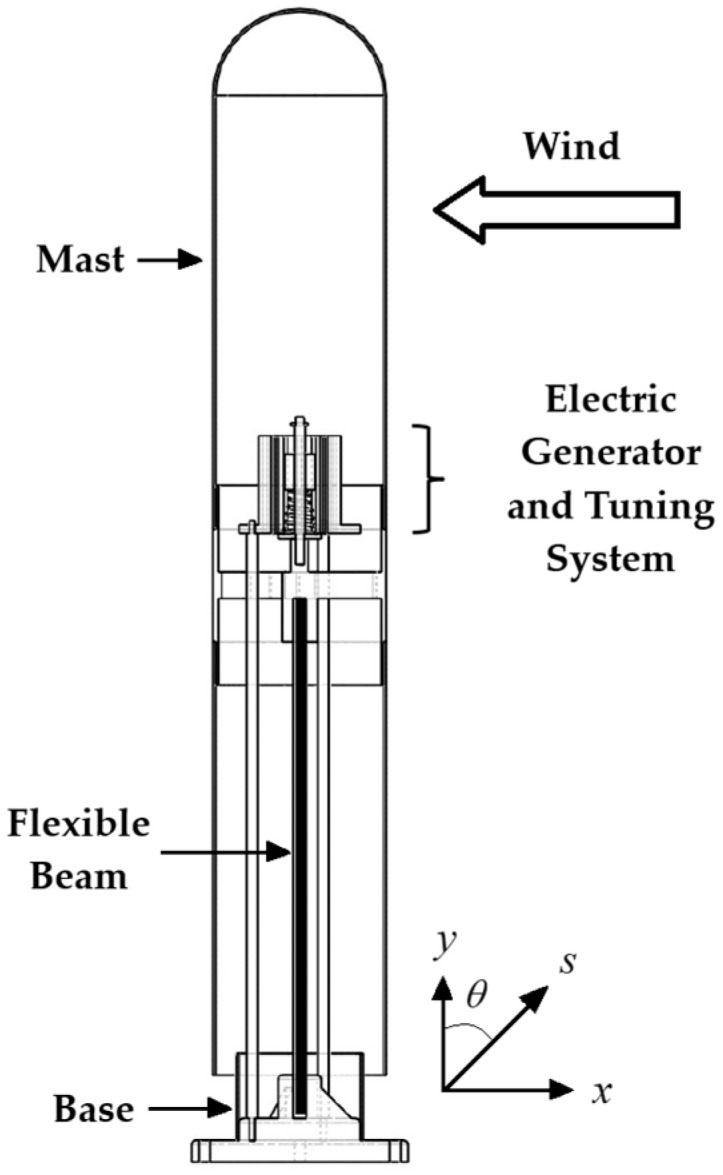
Bladeless wind turbine.

The columnar-cactus type mast is intended for implementation in a VIV bladeless wind turbine mounted at the free end of a flexible beam embedded in the ground (see Figure 1). The flexible beam is a solid circular section made of fiberglass or carbon fiber reinforced polymer. Electric power is generated by a permanent magnet electric generator based on Faraday’s law of induction. In this system, the magnet oscillates, while the coil remains stationary. The electromagnetic induction method has been demonstrated to offer high efficiency in electromechanical energy conversion, energy density, and high voltage [29].

Additionally, the bladeless wind turbine is equipped with an active tuning system that utilizes a non-linear progressive spring to connect the magnet to the mast. The objective of implementing both the tuning system and the columnar-cactus geometry is to maximize power, thereby extending the “lock-in range” where aeroelastic resonance occurs.

## 2. Theoretical Background

The operational principle of these VIV bladeless wind turbines is based on the phenomenon of aeroelastic resonance [30]. This is because there is a transfer of energy from a fluid to a structure that deforms elastically. This aeroelastic phenomenon produces vortex-induced vibrations. For aeroelastic resonance to occur, the natural frequency of the turbine ($f_{n}$) must coincide with the vortex shedding frequency ($f_{v}$). The speed range within which this resonance phenomenon occurs is referred to as the “lock-in range”. Vortex shedding is a phenomenon that occurs when a fluid passes through a structure, typically characterized by a circular cross-section, as the fluid passes, vortices develop; producing lift and drag forces, perpendicular and parallel to the direction of the wind, respectively. The lift forces generate lateral oscillations in the structure at the same frequency as the vortex shedding. This frequency is directly proportional to wind speed. In this manner, oscillations are converted into energy by an electric generator, for example, using the principle of electromagnetic induction.

To optimize the performance of VIV bladeless wind turbines, one or more of the following methods can be implemented. These methods focus on maximizing the resonance phenomenon and, consequently, energy production: (1) geometric and material optimization of the turbine’s primary components [31,32,33,34,35,36,37], e.g., diameter, length, and material of the mast and flexible beam, (2) aerodynamic optimization using vortex generators on the mast [38,39,40,41,42] and (3) optimize turbine operating parameters by implementing tuning systems, e.g., tuning systems with non-linear springs [43], with magnetorheological materials (MERs) [44], with piezoelectric elements [45,46], with magnetic fields [30] and with tuning masses [47] or with the implementation of predictive controllers [48].

The present research relates to the method of aerodynamic optimization using vortex generators. For this reason, the most relevant results in this field are presented below. Ritendra Yadav, et al. [38] developed a bladeless wind turbine with a conical cylindrical mast with side fins attached to the mast, one at 180° from the other. The results demonstrated that this implementation resulted in enhanced energy collection performance at low speeds, while also achieving a 30% reduction in system weight. It is imperative to incorporate a mechanism that enables the turbine’s orientation to be adjusted in accordance with the wind direction. This is because the fins are most effective in extracting energy when positioned perpendicularly to the wind direction.

Adel Younis et al. [39] conducted numerical and experimental studies of a VIV bladeless wind turbine implementing vortex generators on the mast surface. Three scale models of the turbine were used in the study: a simple cylindrical mast, a modified mast with four vortex generators, and another modified mast at full scale with six vortex generators. The experimental tests were carried out in a wind tunnel. In the CFD analysis, the lift coefficients, velocity, pressure, and vorticity contours generated were determined and compared. Regarding the experimental tests, three wind speeds were studied: 10 m/s, 15 m/s, and 20 m/s. Measurements of the converted electrical energy were taken using piezoelectric sensors mounted on the flexible beam of the prototypes at three different heights. The experiments confirmed the CFD numerical results, showing that the full-scale modified mast produced more power. For instance, at a speed of 20 m per second, the power of the simple cylindrical mast was 0.293 nanowatts, whereas that of the full-scale mast was 212 nanowatts.

Anshul Tripathi et al. [40] conducted a study of a VIV bladeless wind turbine with five different mast geometries: circular, decagonal, and three sinusoidal shapes (8, 9, and 10 waves). The study evaluated the modal and structural behavior (static deflection) considering the aerodynamic behavior of the turbine. The flexible rod was affixed to a base, and the mast was exposed to a wind pressure of 15 Pa during the modal analysis. The structural analysis enabled the deflection of the turbine to be calculated under the same pressure produced by the wind. The results demonstrated that sinusoidal shapes exhibited greater deflection, indicating enhanced energy production. For instance, a wind speed of 5 m/s resulted in an increase of 0.5% for an 8-wave sinusoidal mast, 5% for a 9-wave mast, and 8% for a 10-wave mast.

Catherine K. Samy et al. [41] designed a VIV bladeless wind turbine with vortex generators, proposing two designs; the first consists of a conical cylindrical mast with a modified surface into which a spiral longitudinal rib is inserted. This design facilitates simultaneous rotation and oscillation of the mast with respect to direction of the wind. The second design consists of a conical cylindrical mast with vortex generators on its surface. All designs were evaluated based on performance, costs, and portability, and a decision matrix was used to determine the most suitable design. The results demonstrate that these two designs are more economical than conventional turbines and have lower maintenance costs.

Hasan Hamdan et al. [42] performed fluid–structure interaction (FSI) simulations and experimental studies of a VIV bladeless wind turbine. Two mast models with vortex generators on their surface were proposed. The first model is a conical cylindrical mast, with the largest diameter at the top and the smallest diameter at the bottom. The second model is a cylindrical mast with double conicity, where the largest diameter is located at half its length, and the smallest diameters are located at the top and bottom of the mast. The vortex generators were strategically positioned along the surface of the mast, ensuring comprehensive coverage. These vortex generators are implemented to improve the vortex shedding effect and vorticity formation around the turbine. The findings include the identification of optimal vibration frequencies and amplitudes that improve energy capture, as well as a clear advantage in energy generation estimates. The second model demonstrated superior aerodynamic performance in comparison to the first, as evidenced by the enhancement in vortex shedding frequency and energy generation estimates.

## 3. Mathematical Treatment

In this research, a VIV bladeless wind turbine consisting of a cylindrical mast operating on the principle of vortex-induced vibration was selected with the aim of maximizing its aerodynamic performance. This was achieved by implementing the cross-sectional geometries of the mast with columnar cactus geometries, as these geometries are vortex generators. When air fluid flows the circular mast, low-pressure vortices form downstream of the mast on both sides. These vortices are periodic and depend on the Reynolds number (Re), which is determined by the following equation [44]:

(1) $$Re=\frac{\rho_{a}U_{f}D_{m}}{\mu }$$

where $\rho_{a}$ is the air density, $U_{f}$ is the wind speed, $D_{m}$ is the mast diameter, and μ is the dynamic viscosity of the air. The Karman vortex street, which causes differences in air fluid pressure on the surface of the mast, generates forces that are also periodic due to the detachment of these vortices. These forces are perpendicular to the direction of the wind and change direction depending on the appearance of the vortices. These forces are called lift forces. The lift force is periodic at the vortex shedding frequency, which depends on the wind speed and the diameter of the cylinder and is determined by the following equation [43]:

(2) $$F=\frac{1}{2}\rho_{a}U_{f}^{2}D_{m}L_{m}C_{L}sen(2\pi f_{v}t)$$

where $L_{m}$ is the length of the mast and $C_{L}$ is the lift coefficient, which has a value of 0.50 for Reynolds numbers between 10^3^ < Re < 10^5^ [49]. The vortex shedding frequency (Hz) is determined by the following equation [43]:

(3) $$f_{v}=\frac{S_{t}U_{f}}{D_{m}}$$

where $S_{t}$ is the Strouhal number (approx. 0.20, [50]) and represents a measure of the ratio of the inertial forces of the flow and the inertial forces of convective acceleration. When the frequency due to vortex shedding coincides with the natural frequency of the turbine, i.e., $f_{v}$ ≈ $f_{n}$, the aeroelastic resonance of the system produces large-amplitude vibratory oscillations. The wind speed range ($U_{lock}$) that causes this aeroelastic resonance is known as “lock-in range” and can be approximated as [44]:

(4) $$U_{lock}\approx \frac{D_{m}}{S_{t}}\left(f_{n}\right)$$

Even if the vortex shedding frequency does not exactly match the natural frequency of the turbine, the turbine will continue to operate under aeroelastic resonance conditions if the wind speed is within this “lock-in range”. The VIV bladeless wind turbine is a simple structure that can be modeled as a forced mass-spring-damper system with a tuning system. The natural frequency of the turbine can be determined using the following equation [30]:

(5) $$f_{n}=\frac{1}{2\pi }\sqrt{\frac{\left(k+k^{\prime }\left(x\right)\right)}{m}-{\left(\frac{c}{2m}\right)}^{2}}$$

where *k* and *c* are the stiffness and damping of the flexible beam, respectively, *k*′(*x*) represents the amount of elasticity provided by the tuning system, and m is the mass of the mast. Therefore, given the design criteria for wind speed and mast diameter, these parameters are carefully selected to meet the “lock-in range” condition for the vortex shedding frequency. The stiffness of the flexible beam *k* can be determined using the following equation [43]:

(6) $$k=\frac{3\pi }{64}\frac{Ed^{4}}{L^{3}}$$

where d and L are the diameter and length of the flexible beam, respectively, and E is the modulus of elasticity of the flexible beam material.

## 4. Methodology

This section describes the methodology employed in this research. First, the morphology of the columnar cactus is briefly characterized, emphasizing how its ribbed geometry promotes cooling through the generation of air currents along the stem sur-face. Subsequently, the design and fabrication of a scaled prototype of the bladeless wind turbine are detailed. The columnar cactus geometries were implemented in the mast design, and their performance was compared with that of a conventional cylindrical mast to validate the proposed hypothesis. Next, the development of a custom load cell is presented. This device was designed to measure the vortex-induced vibrations generated in each prototype using strain gauges. A mounting system was also designed to secure the load cell and to isolate the vibrations induced by the prototypes from external disturbances. Finally, the characteristics of the wind tunnel used for testing are described, along with the determination of the corresponding test velocity range.

### 4.1. Morphology of the Columnar Cactus

The subfamily “Cactoideae” within the family “Cactaceae” includes the columnar cactuses. These plants are characterized by the absence of leaves or glochidia, and they are grouped into six tribes. The families in question are: Browningieae, Calymmantheae, Cereeae, Notocacteae, Pachycereeae, and Trichocereeae [51]. Columnar cactuses are endemic to the tropical and subtropical regions of the American continent, with notable diversity found in Brazil, Mexico, and Peru [51]. In Mexico, there are approximately 70 species of columnar cactuses, 45 of which are found on the southern Pacific slope, which includes the Tehuacán Valley and the Balsas Depression. This region is widely recognized as a global leader in the diversity of columnar cactuses, boasting an unparalleled variety that is unrivaled worldwide [51].

The following are some of the main characteristics of columnar cactuses [52]: Elongated, cylindrical, and dense stems with vertical V-shaped ribs. The number of vertical ribs and their size can vary significantly, ranging from a few centimeters to several meters in height [53]. The vertical ribs of columnar cactuses serve several key functions, including structural strength, water storage, and temperature regulation [54]. The ribs, which are longitudinal ridges on the stem, help make the cactus more resistant to bending and the wind’s forces, especially in arid climates. Additionally, these ribs enable the cactus to expand and contract as it absorbs and releases water, a process vital to its survival in arid environments.

**Figure 2:**
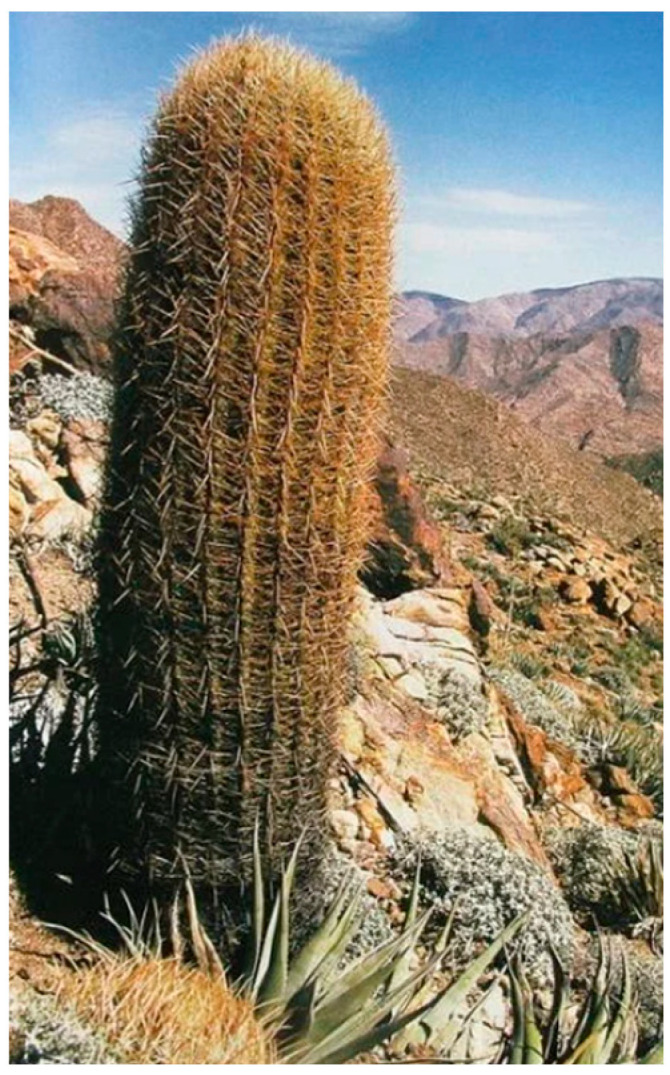
Barrel cactus (*Ferocactus acanthodes*), Sierra de Juárez, Baja California, Mexico [58].

**Figure 3:**
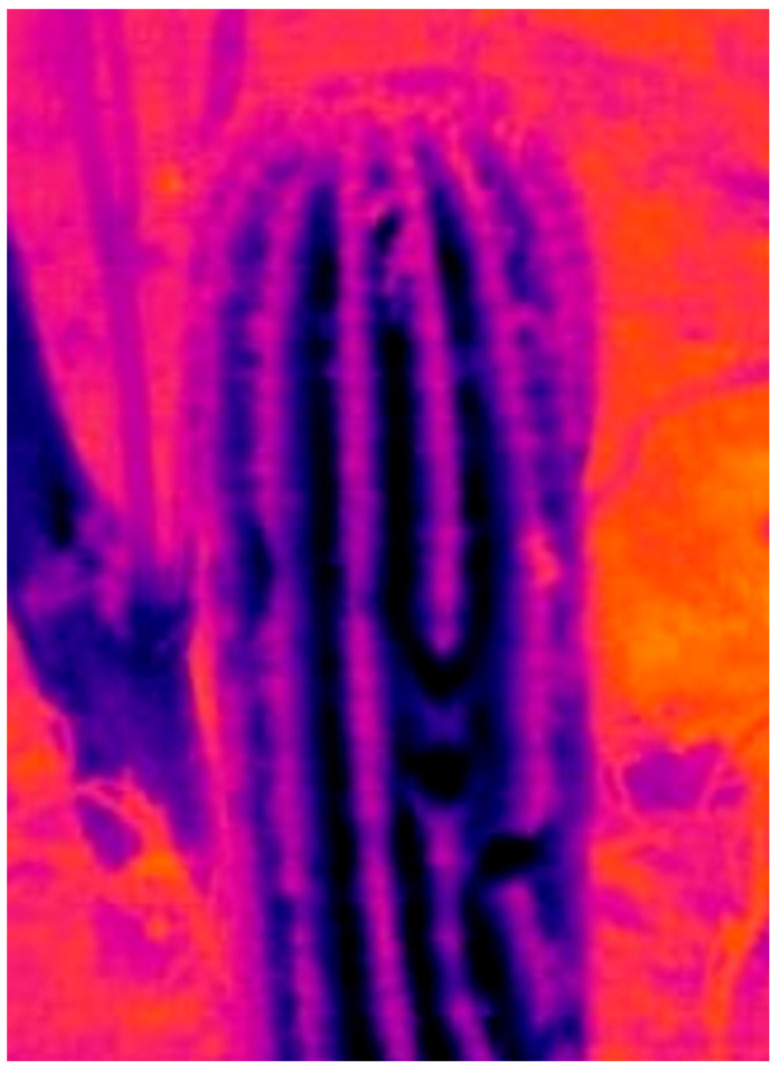
Infrared image of a cactus [54].

The configuration of the ribs generates a surface that mitigates the amount of direct sunlight reaching the stem. Additionally, when wind encounters the ribs, it generates turbulent airflow patterns that reduce the cactus temperature. This phenomenon has been thoroughly studied in the research conducted by Donald A. Lewis and Park S. [55]. They conducted an energy balance study, during which they examined latent heat, as well as heat transfer by radiation, conduction, and convection in a barrel cactus (*Ferocactus acanthodes*). They developed a mathematical model and conducted experiments, studying the thermal behavior on the surface of the cactus during a full day under summer and winter weather conditions. Among the most significant findings was the identification that the ribs produced a convective coefficient per unit area 67% higher than a smooth cylinder of the same outer diameter and height, which represented a reduction in the average daytime surface temperature of 5 °C. These studies were conducted under field conditions. As shown in Figure 2, the barrel cactus (Cactaceae) is a member of the Cactaceae family. Figure 3 presents an infrared image of a cactus. This figure illustrates how the valley areas of the cactus experience cooler temperatures due to the shade and air currents produced by the ribs.

**Figure 4:**
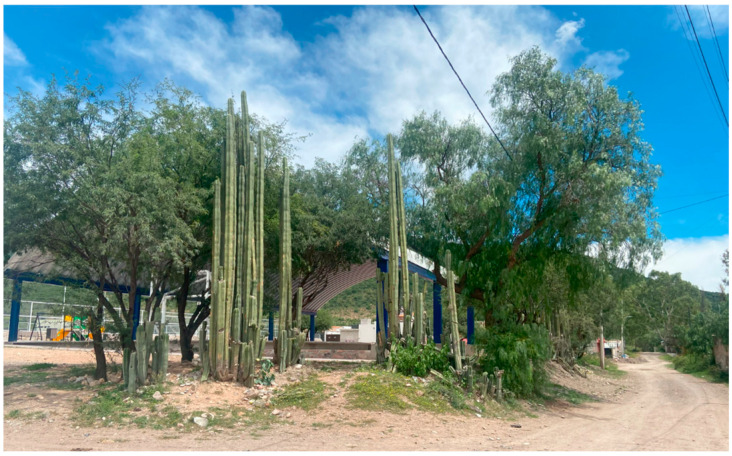
Group of “Chilayo” columnar cactuses in the municipality of El Marqués, Querétaro, Mexico.

This research works with the geometry of the cactus known as “chilayo”, “organ cactus,” or “jarritos” (Lophocereus marginatus), which belongs to the tribe “Pachycereeae” as this cactus is primarily distributed in central Mexico. For example, in the state of Querétaro, it is present in the municipalities of Arroyo Seco, Jalpan de Serra, El Marqués, Colón, Tolimán, Cadereyta, Ezequiel Montes, and Tequisquiapan [56]. The “chilayo” cactus, native to Mexico, can reach heights of 3 to 5 m. It possesses a straight, dark green stem with a diameter of 8 to 15 cm and 4 to 7 prominent vertical ribs [57]. As illustrated in Figure 4, a group of “chilayo” cactuses is located in the municipality of El Marqués, Querétaro, Mexico.

### 4.2. Prototype Design with Cylindrical Mast

The first step in conducting the experimental studies is to size the scaled prototype of the VIV bladeless wind turbine. These studies exclusively address the mast, the component responsible for capturing wind energy. The mast is a straight cylindrical structure with a nominal diameter of 28 mm, coupled to the free end of a flexible beam of fiberglass-reinforced polymer ($E_{1}=29\;\mathrm{GPa}\;[\mathrm{Young}’s\;\mathrm{modulus}\;0{}^{\circ}],\;\rho =2100\;\mathrm{kg}/m^{3}$). The beam has a diameter of 5 mm and an effective length of 160 mm. The other end of the flexible beam is connected to a “load cell” of our own design, which in turn is fixed to the fixture located in the wind tunnel test area. A length of 20 mm is considered at each end of the flexible beam for attachment to the mast and “load cell”. The mast is hollow, with a thickness of approximately 3 mm, but has a solid core for connection to the flexible beam. This core allows us to adjust the mass of all prototypes with different cactus geometries. The variations in vortex-induced vibrations are only due to aerodynamic effects and not to variations in the natural frequencies that depend on their mass and the effective length of the flexible beam.

**Figure 5:**
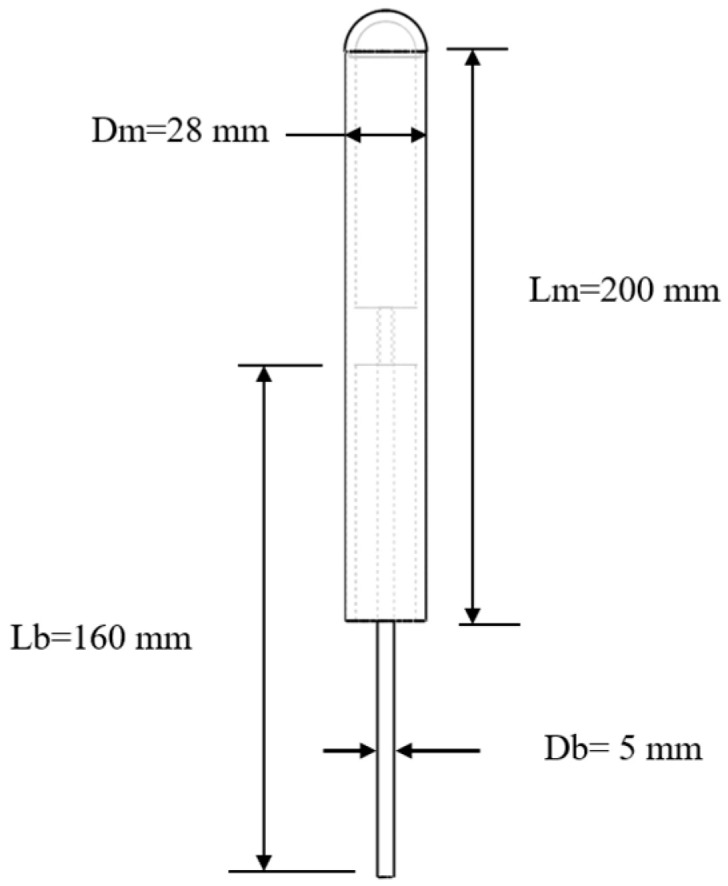
Dimensions of the scaled prototype of the turbine with cylindrical mast.

The configuration and dimensions of the scaled prototype of the turbine with a cylindrical mast are presented in Figure 5. These dimensions align with the prototypes presented in research [34,59], considering the capabilities of the wind tunnel. This approach facilitates the execution of comparative experimental studies in the future. The aerodynamic optimization of the bladeless wind turbine focuses on the geometric characteristics of the mast, including its dimensions and shape, to facilitate vortex generation and separation, thereby promoting vortex-induced vibration (VIV).

### 4.3. “Load Cell” Design

The “load cell” has an “I” shape, with its web thickness aligned with the main plane of oscillation so that mechanical strains can be recorded along its length. The “load cell” is instrumented with two strain gauges that measure strain (με or 1 × 10^−6^ m/m) or voltage changes (m *v*/*v*). A strain gauge is placed on each side of the web thickness, and both are strategically positioned to ensure optimal performance. They are located as close to the bottom of the “load cell”, where maximum deflections occur, since these regions are farthest from the mast where wind-induced forces act. This positioning maximizes the signal produced by the strain gauges, ensuring high-quality data collection. The purpose of installing strain gauges on both sides of the “load cell” is to measure the same strain signal but with opposite signs. In other words, while one-gauge measures strains in tension, the other measures strain in compression. If both strain gauges record the same magnitude, this indicates that the scaled turbine prototype is properly aligned with the wind direction and that the bonding and installation of the strain gauges were adequate.

The strain gauge measurements are related to the forces induced by the wind. These forces consist of lift forces acting in the plane perpendicular to the wind direction and the inertial forces of the mast. It is important to note that the primary objective of this study is not to determine the exact magnitude of the wind-induced forces, but rather to assess the variation in these forces resulting from different mast geometries. To this end, strain measurements corresponding to “load-cell” deflections are obtained using strain gauges under vortex-induced vibration conditions. Additionally, a data acquisition system is used to monitor both the strain on the “load cell” and the wind speed in real time.

**Figure 6:**
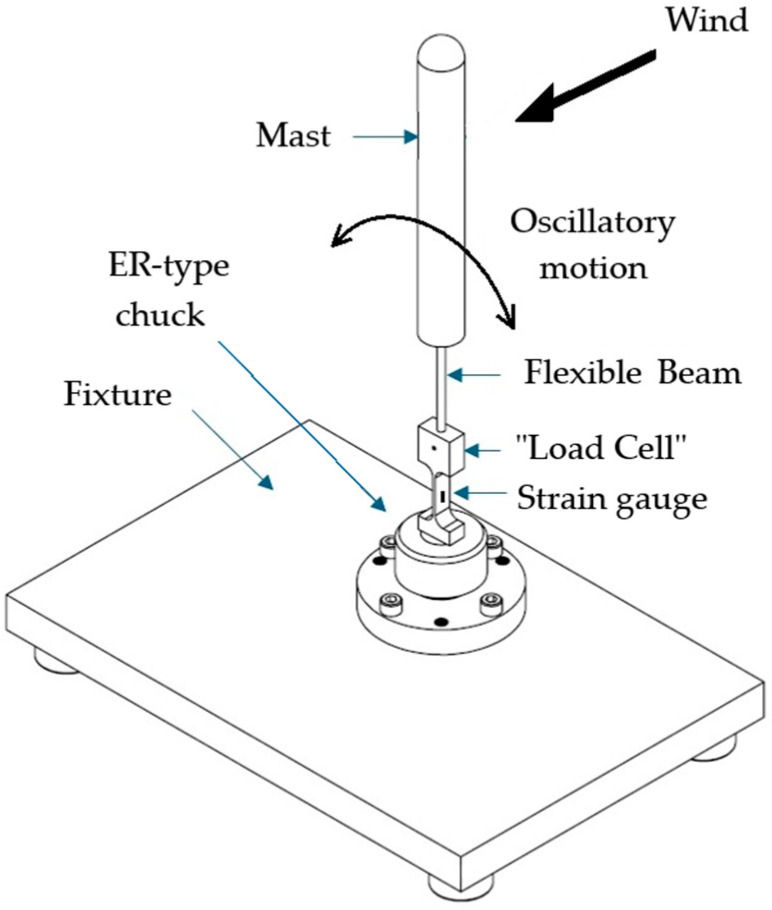
Assembly of the “load cell” with the flexible beam and mast on the fixture.

A special fixture was also designed and manufactured to secure the “load cell”. The “load cell” is firmly attached to the fixture using a self-locking ER-type chuck, ensuring stability during testing. The fixture provides high rigidity relative to the mast–flexible beam–“load cell” assembly, thereby ensuring noise-free measurements. It is equipped with heel-type dampers, which prevent vibration transmission between the wind tunnel and the assembly. As illustrated in Figure 6, the “load cell” is to be assembled with the flexible beam and the mast on the fixture.

The strain gauge used was model CEA-06-240UZ-120, and the data acquisition system model was MM01-120, both manufactured by Micro Measurements from North Carolina, USA [60]. The selection of measurement equipment prioritized an acquisition frequency of 80 Hz. The system records data in units of strain (µε) and/or voltage (m *v*/*v*), with internal bridge termination supporting full-bridge, half-bridge, and quarter-bridge configurations. The data were stored in *.CSV format on a USB memory device for subsequent processing and analysis.

### 4.4. Design of Columnar-Cactus Type Mast Prototypes

To expand the study and test the hypothesis, three prototypes with modified geometries were tested in addition to the cylindrical mast prototype, which has a diameter of 28 mm. The prototypes feature a columnar-cactus type geometric shape with 5, 6, and 7 ribs, also with a nominal diameter of 28 mm. During the sample collection process, no 4-rib cactuses were identified, leading to the exclusion of this cactus-type mast from experimental studies.

**Figure 7:**
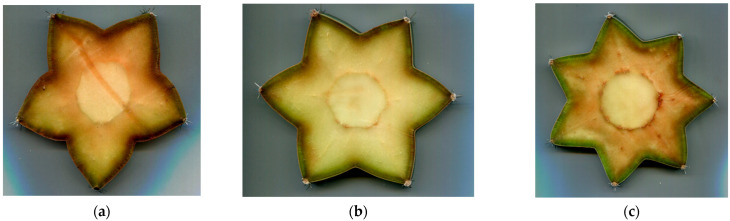
Cross-sections of columnar cactuses. (**a**) 5 ribs, (**b**) 6 ribs, and (**c**) 7 ribs.

**Figure 8:**
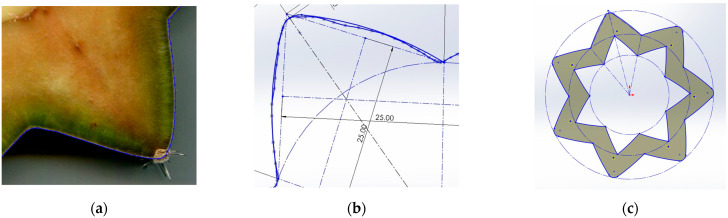
Process for CAD modeling of columnar-cactus mast profiles. (**a**) “spline” command for the contour, (**b**) replacement of “spline lines” with “arc lines”, and (**c**) scaling and “equidistant entities”.

The process for determining the cactus geometries was as follows: a cross-section was taken from each columnar cactus with a different numbers of ribs (see Figure 7), these segments were scanned using a flatbed scanner, and the resulting images were imported into SolidWorks^®^ 2024 software, where the profile or outline of each cactus geometry was generated using the “Spline” command (see Figure 8a). The “Arc” command was then applied to replace the spline curves with arcs, facilitating Computer-Aided Design (CAD) processing and prototype manufacturing (see Figure 8b). It was essential to ensure that this replacement process did not alter the original cactus geometry. The cactus profile was then scaled to a nominal diameter of 28 mm, ensuring that all dimensions remained inscribed within this diameter. Finally, the “Equidistant Entities” command was used to provide wall thickness to the cactus body (see Figure 8c).

**Figure 9:**
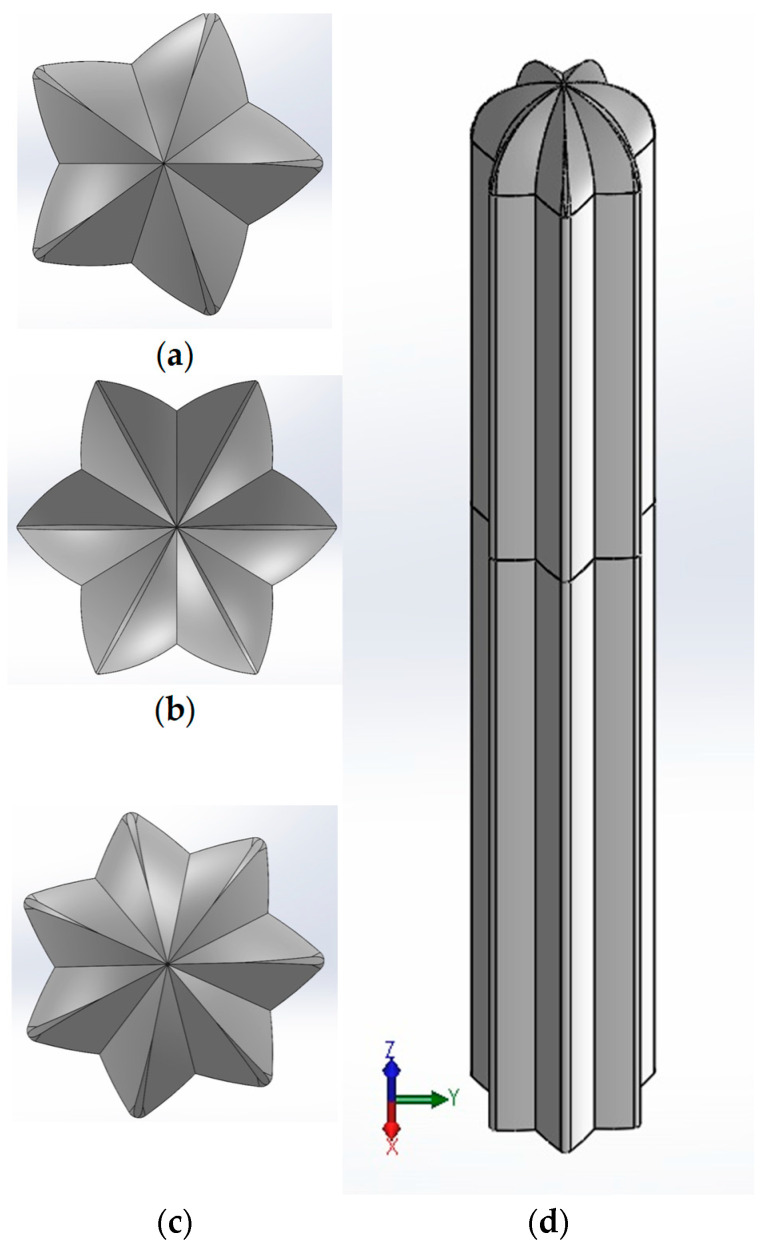
Profiles of cactus-type masts. (**a**) 5 ribs, (**b**) 6 ribs, (**c**) 7 ribs and (**d**) assembled model of the 7 ribs cactus-type mast.

The thickness of the material can be adjusted to accommodate variations in the geometries of the cactuses. This adjustment is achieved through CAD modeling, which is used during the manufacturing process of the prototypes. Figure 9 shows the profiles of the cactus-type masts modeled in SolidWorks^®^ 2024 software: 5 ribs (Figure 9a), 6 ribs (Figure 9b), and 7 ribs (Figure 9c). Each cactus-type mast prototype was divided into three segments. The first segment corresponds to the lower part of the mast and includes the core where it connects to the flexible beam. The middle segment is an extension of the mast, while the upper segment forms a dome or cupola with the same geometry as the cactus, converging along the mast’s central axis. Figure 9d shows the assembled cactus-type mast prototype with 7 ribs. The prototypes were segmented according to the requirements for assembling the mast with the flexible beam. The joints between segments feature male-female extensions to ensure proper collinearity between parts.

### 4.5. Study of the Natural Frequencies of Prototypes

**Figure 10:**
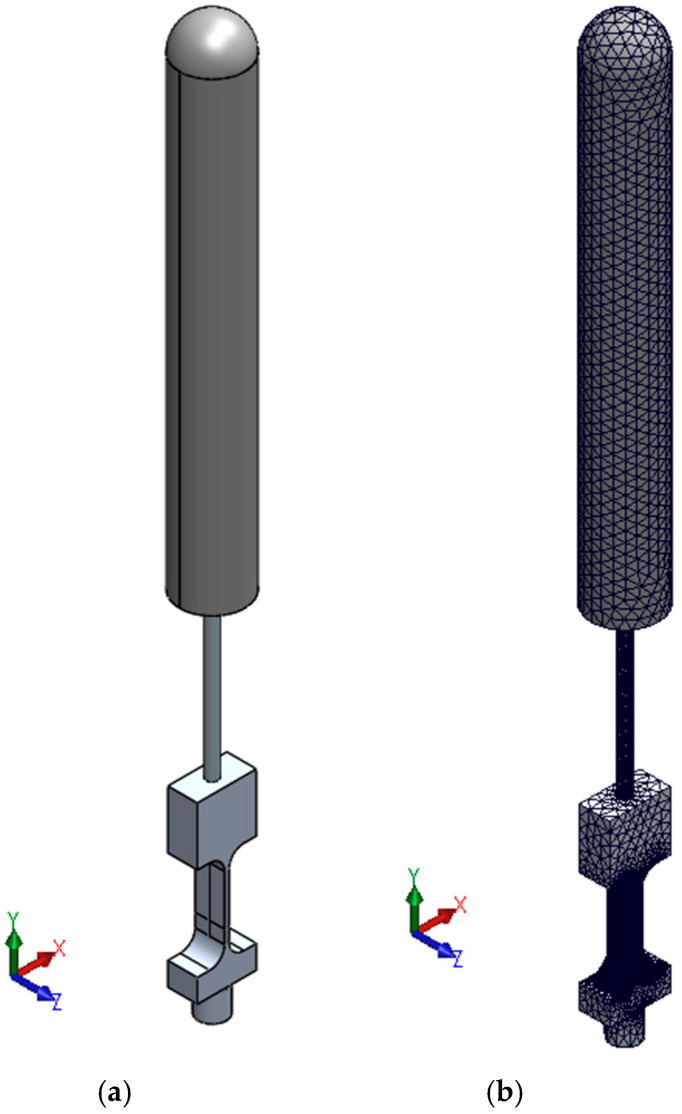
Mast–flexible beam–“load cell” assembly. (**a**) 3D CAD model, (**b**) finite element model.

Before proceeding with the manufacturing of the prototypes, a natural frequency analysis was conducted on the cylindrical mast–flexible beam–“load cell” assembly (see Figure 10a). This analysis was performed to determine the magnitude of its first five natural frequencies and corresponding vibration modes. The objective was to predict the dynamic behavior of the assembly and to confirm that the wind tunnel’s operational range was adequate for testing. As illustrated in Figure 10b, the finite element model (FEM) of the assembly is shown. The model consists of 908,684 elements. A mesh convergence analysis was carried out to minimize or eliminate potential inaccuracies and errors in the results that could arise from the mesh size. The lower part of the “load cell” was constrained in all degrees of freedom.

**Figure 11:**
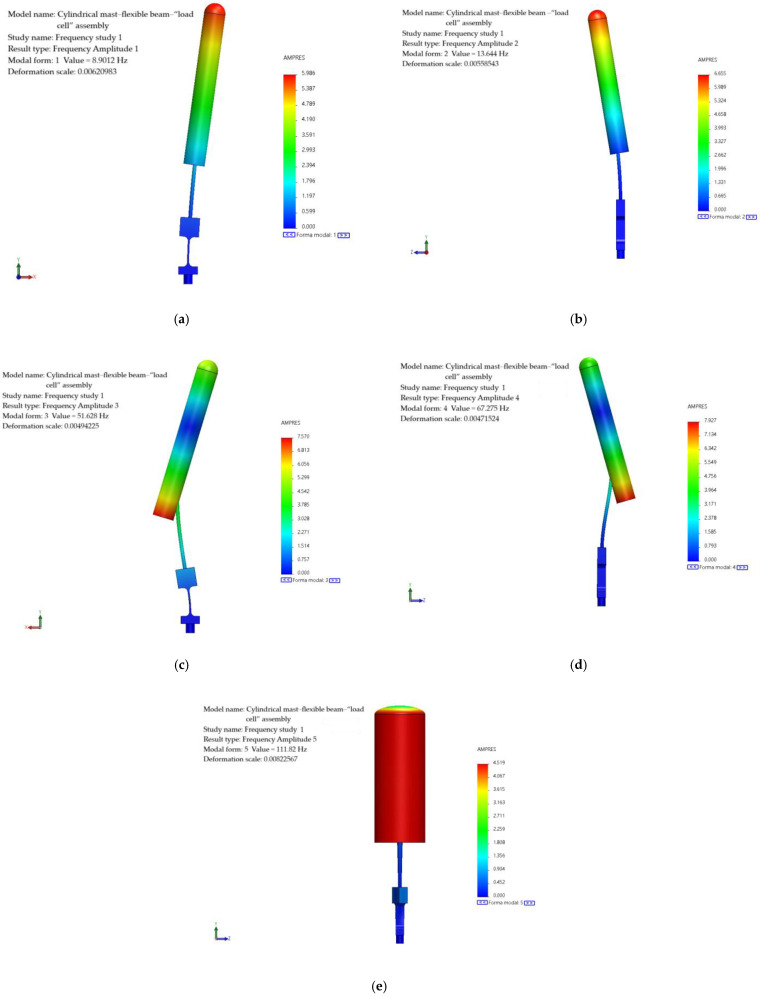
Natural frequencies of the mast–flexible beam–“load cell” assembly. (**a**) First frequency, (**b**) second frequency, (**c**) third frequency, (**d**) fourth frequency, and (**e**) fifth frequency.

As illustrated in Figure 11, the first five natural frequencies and their associated vibration modes are presented. As illustrated in the figure, the first and third frequencies are associated with oscillatory motion in the plane transverse to the wind direction. However, the first frequency has a pivot node at the bottom of the “load cell”, while the third frequency has the pivot node near the connection between the mast and the flexible beam. In a similar manner, the second and fourth frequencies demonstrate oscillatory motion, but in a plane that aligns with the wind. It should be noted that the second frequency has its pivot node situated at the base of the “load cell”, while the fourth frequency features the pivot node near the point of connection between the mast and the flexible beam. The fifth frequency exhibits a torsional mode with respect to the axial axis of the flexible beam.

Table 1 presents the initial five natural frequencies of the assembly, the wind speed, and the associated Reynolds number. During the testing phase, an increase in the magnitude of vortex-induced vibration would be expected to occur in proximity to these associated wind speeds due to aeroelastic resonance.

### 4.6. CDF Modeling of Vortex Shedding of Bladeless Wind Turbine Prototypes

Before proceeding with the manufacturing of the prototypes, a CFD analysis of vortex shedding was performed for both the cylindrical mast and the 5-rib cactus-type mast. This simulation represented the initial step in evaluating the hypothesis proposed at the beginning of the research project. In this simulation, air was designated as the free-flowing fluid, and its properties at sea level are summarized in Table 2.

These simulations were performed for a Reynolds number range of 8000 to 30,000, which falls within the capacity of the wind tunnel where the prototype tests were conducted. A two-dimensional (2D) flow model was used, with forces induced by the transverse flow acting on each section of the structure. This simplification has proven effective, yielding satisfactory results [32]. The diameter of the straight cylindrical mast was 0.028 m, and the free stream fluid velocities corresponding to the Reynolds numbers mentioned above ranged from 3 to 10 m/s, respectively.

**Figure 12:**
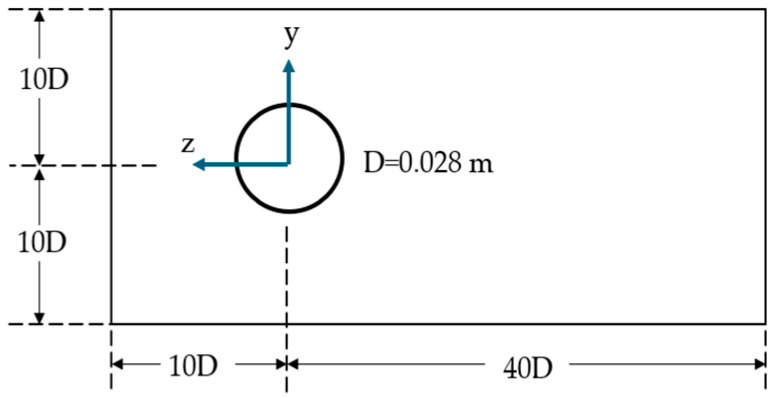
CFD numerical model domain.

Figure 12 shows the computational domain constructed to simulate the scaled prototypes of the bladeless wind turbine. This domain was modeled as a numerical study of flow passing over a stationary 2D cylinder using the k–ε turbulence model. The upper and lower boundary dimensions were set to 10D, while the left and right boundaries were set to 10D and 40D, respectively.

**Figure 13:**
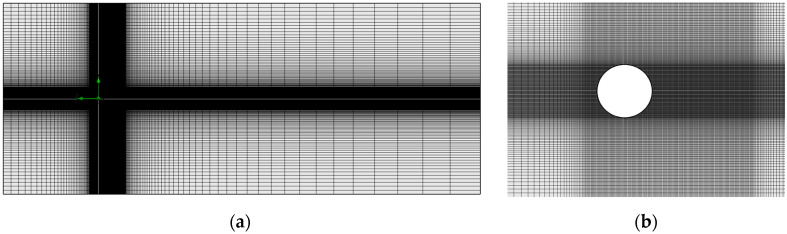
Mesh generated from the domain. (**a**) Entire domain and (**b**) mesh around the turbine.

The simulation process was carried out using a structured quadrilateral mesh implemented in SolidWorks^®^ 2024 Flow Simulation software (see Figure 13). The control parameters for the numerical simulation were as follows: transient analysis type (time-dependent), external fluid flow, laminar and turbulent flow, adiabatic walls, turbulence intensity of 0.1%, turbulence length of 0.0001 m, time step of 0.001 s, and analysis duration of 10 s.

To ensure the reliability of the results, a mesh sensitivity analysis was performed at the outset. This step ensured that the results would not be affected by mesh resolution, thereby reducing or eliminating potential inaccuracies and errors caused by mesh size. In this numerical study, the mesh sizes analyzed were 20k, 40k, 60k, and 80k for a Reynolds number of 23,000.

**Figure 14:**
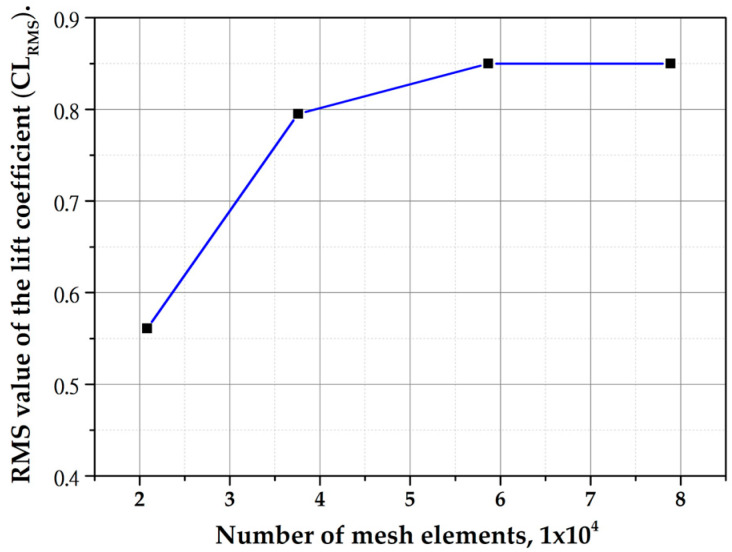
RMS value of the lift coefficient (CL_RMS_) as a function of the number of mesh elements.

The root mean square (RMS) value of the lift coefficient (CL_RMS_) was used as a mesh convergence parameter. The RMS value quantifies the intensity or magnitude of parameter fluctuations around their mean value. The objective was to determine the minimum mesh size that would yield accurate and reliable results while minimizing computational costs. Once this parameter stabilizes, it can be concluded that the mesh convergence process is complete. As illustrated in Figure 14, the RMS value of the lift coefficient (CL_RMS_ = 0.85) stabilizes when a mesh of 60,000 elements is employed. Therefore, this mesh size was selected to calculate the lift coefficient.

This study presents the RMS value of the lift coefficient (CLRMS) for both the cylindrical mast and the 5-rib cactus-type mast. This parameter is used to assess the relative aerodynamic efficiency of each mast, as the lift coefficient is associated with the amplitudes of vortex-induced vibrations and, consequently, with the conversion of wind energy into usable power.

**Figure 15:**
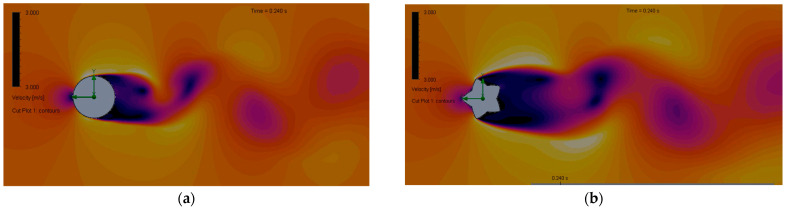
Vortex shedding. (**a**) Cylindrical mast and (**b**) 5-ribs cactus-type mast.

As illustrated in Figure 15, the results of the vortex shedding simulation for both masts are presented. The figure shows a wider von Kármán vortex street for the 5-rib cactus-type mast, indicating increased vortex-induced vibration amplitude and, consequently, a higher lift coefficient. This increase in bandwidth occurs because the cactus ribs promote the formation and shedding of vortices.

**Figure 16:**
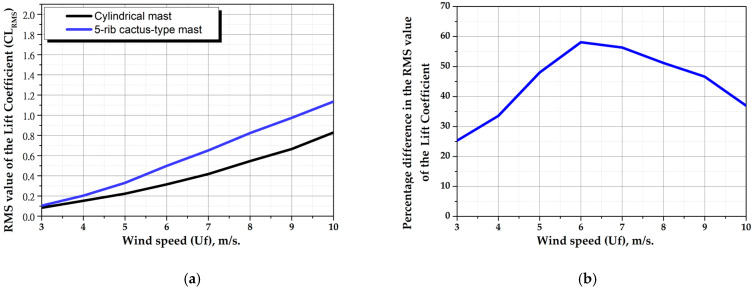
Results of CFD analysis. (**a**) RMS value of the lift coefficient (CL_RMS_) on both masts, (**b**) percentage difference between the two masts.

Figure 16a shows the RMS value of the lift coefficient of the cylindrical mast and the 5-ribs cactus-type mast as a function of wind speed. This figure shows that the 5-ribs cactus-type mast develops greater lift coefficient at all wind speeds. Figure 16b shows the percentage difference in lift coefficient of both masts as a function of wind speed. For example, at a wind speed of 6 m/s, the difference is close to 60%. This information contributes significantly to the verification of the hypothesis. It is important to mention that this CFD analysis only includes the aerodynamic study of vortex shedding and does not consider the fluid–structure interaction of the turbine.

### 4.7. Manufacturing of Columnar-Cactus Type Masts Prototypes

**Figure 17:**
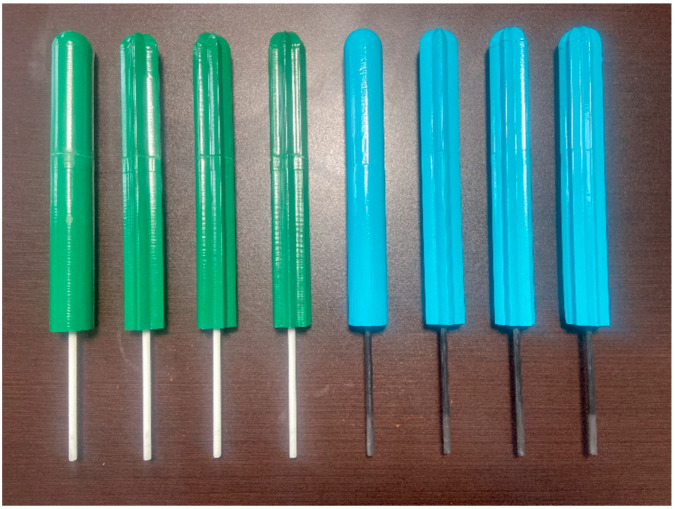
Cactus-type masts with a flexible beam of the fiberglass-reinforced polymer (white) and with a flexible beam of the fiber carbon-reinforced polymer (black), from left to right: cylindrical mast, 5-ribs cactus-type mast, 6-ribs cactus-type mast, and 7-ribs cactus-type mast.

Following the CFD numerical analyses, the prototypes were manufactured and assembled. These prototypes were printed using the FDM (*Fused Deposition Modeling*) 3D printing technique with PLA-FC carbon fiber material. The masts were attached to the flexible beams using a professional-grade epoxy adhesive, ensuring the collinearity and effective length of the flexible beam. The surfaces of the masts were prepared with automotive paste to fill the porosity resulting from the printing process and then coated with primer and acrylic enamel paint. As illustrated in Figure 17, eight cactus-type masts were produced: the first four with flexible beams made of fiberglass-reinforced polymer (white), and the remaining four with flexible beams made of carbon fiber–reinforced polymer (black). From left to right, the sequence includes the cylindrical mast, the 5-rib cactus-type mast, the 6-rib cactus-type mast, and the 7-rib cactus-type mast.

The manufacturing and assembly of the prototypes were carried out under strict quality control measures to ensure that any differences in the behavior of the masts were solely attributable to the geometric shapes of their cross-sections, and not to variations in the mass or effective length of the flexible beams that could alter the results by changing the natural frequencies of the prototypes. The mass of each prototype is listed in Table 3. The maximum difference among the prototypes is 0.37 g, which corresponds to 0.48% of the total mass. It will be demonstrated subsequently that this variation has no significant effect on the natural frequencies of the prototypes.

### 4.8. Wind Tunnel Preparation

**Figure 18:**
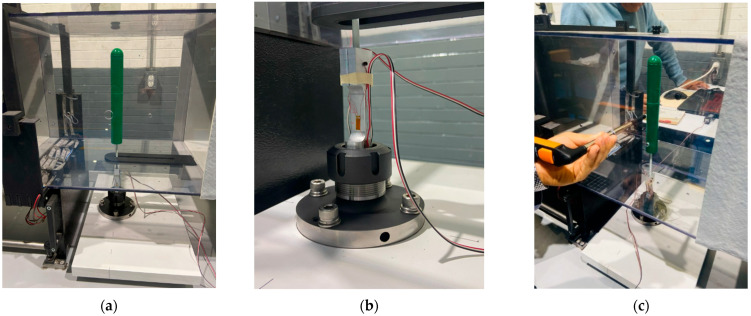
Wind tunnel preparation. (**a**) Assembly of the mast–flexible beam–“load cell” assembly, (**b**) detail of the instrumented “load cell” and (**c**) hot-wire anemometer for measuring tunnel wind velocity in the wind tunnel.

The tests were carried out in the HM 170 wind tunnel, manufactured by G.U.N.T. Gerätebau GmbH from Barsbüttel, Germany [61] located at the Instituto Tecnológico de Querétaro, it is an open wind tunnel “Eiffel” type, has a working section of 292 × 292 mm and a length of 420 mm. The tunnel wind velocity can be adjusted with a frequency variator to a range of 2.5 to 25 m per second. In these experiments, the term “wind speed” and “tunnel wind velocity” are synonymous. As illustrated in Figure 18a, the mast–flexible beam–“load cell” assembly is mounted within the wind tunnel test area. The mast and flexible beam are aligned with the “load cell”, which is aligned with the fixture, and the fixture is aligned with the working area of the tunnel. As shown in Figure 18b, the “load cell” is mounted on the self-locking ER type nozzle mandrel. The experimental speed range for the test was determined based on the operating conditions and limitations of the wind tunnel. The tunnel wind velocity is measured using a testo 405i hot-wire anemometer manufactured by Testo SE & Co. KGaA from Titisee-Neustadt, Germany [62].

A CFD analysis was conducted on the scaled prototype to rule out significant wall and blockage effects in the wind tunnel due to the dimensions of the prototype. The CFD domain corresponds to the test volume of the wind tunnel, and a windspeed of 3.0 m/s was used. The length between the upper cover of the prototype and the wind tunnel top cover is 2.0 Dm (twice the diameter of the prototype). It was identified that from 1.0 Dm onwards, the fluid streamlines no longer show significant effects, i.e., the streamlines remain uniform under laminar flow conditions. This CFD analysis also allowed determine the most suitable position for the hot-wire anemometer to measure wind speeds during testing, due to space limitations and difficulty in taking readings upstream of the scaled prototype. The length between the prototype and the side walls of the wind tunnel is 5.0 Dm. The CFD analysis findings determined that, starting at 2.0 D with respect to the scaled prototype, the fluid streamlines do not show significant effects. Therefore, the hot-wire anemometer was located 3.5 D from the prototype and 1.5 D from one of the side walls of the wind tunnel (see Figure 18c). In addition, measurements were taken upstream of the prototype and from the selected position, and the results showed a difference of less than 5%, which allowed the selected position to be validated.

## 5. Results

The natural frequency of each prototype is determined by the mass distribution of the mast and the stiffness of the flexible beam. Although the variation in mass presented in Table 3 is less than 0.5% between the lightest and heaviest prototypes, and the effective length of the flexible beam remained constant during assembly, it is necessary to experimentally measure the natural frequency of each prototype to ensure that the variations in vortex-induced vibration are due to aerodynamic effects and not to changes in the natural frequency.

**Figure 19:**
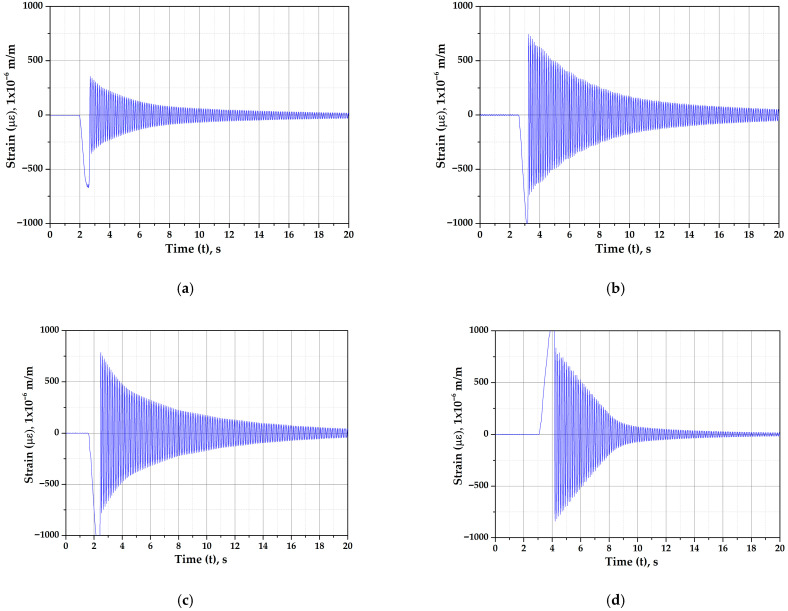
Natural frequencies of the masts. (**a**) Cylindrical, (**b**) 5-ribs cactus-type, (**c**) 6-ribs cactus-type, and (**d**) 7-ribs cactus-type.

The procedure for determining the natural frequency was as follows: First, the mast was subjected to a hammer blow or manually moved perpendicular to the direction of the wind and then released abruptly. As a result of this disturbance, the damp response was obtained through the strain gauges (see Figure 19). Using the information obtained from the graphs, the logarithmic decrement (δ) was determined by measuring two adjacent oscillations, or any two oscillations, while accounting for the number of cycles between the selected oscillations. The logarithmic decrement allows for the calculation of the damping ratio (ζ), which in turn enables the determination of the damped natural frequency (ω_d_). The natural frequency (ω_n_) is derived from the damp frequency and the damping ratio. Finally, the linear frequency (f_n_) is obtained, which is related to the natural frequency by the constant 2π.

Table 4 presents the experimental natural frequency values for each prototype, which correspond to their first fundamental frequencies. As can be seen, the frequencies are very similar to each other, which was to be expected since the mass and stiffness of the flexible beam, which is related to its effective length, were controlled during the manufacturing and assembly process.

A comparison between the experimentally determined natural frequencies (8.33–8.25 Hz) and the first natural frequency obtained numerically (8.9 Hz) reveals a deviation of 6.4–7.3%, respectively. This deviation is primarily attributed to the mechanical properties of the flexible beam considered in numerical structural analysis, as these properties were obtained from literature. However, the maximum deviation from the experimental natural frequency was only 1.02%, confirming that the magnitudes of the vortex-induced vibrations depend solely on the aerodynamic behavior determined by the prototype geometry and not on structural aspects of the components. The geometry of the prototype is a fundamental factor in determining its natural frequency. Therefore, the initial natural frequency is used to determine the wind speed (U_lock_) at which vortex-induced vibrations occur. This is achieved by matching the natural frequency of the structure with the vortex shedding frequency.

The wind speed at which aeroelastic resonance occurs was calculated using Equation (4), resulting in a value of 1.162 m/s ($D_{m}$ = 0.028 m, $f_{v}$ = 8.3 Hz, and $S_{t}$ = 0.2). However, the wind tunnel operates from 2.5 m/s due to the atmospheric conditions of the site (1820 masl), which limits the study of natural frequencies below this wind speed. Therefore, when considering the numerical frequency results, the third and fourth fundamental frequencies can be studied. These frequencies are associated with oscillatory vibration modes, with values between 51.62 and 67.27 Hz, corresponding to wind speeds of 7.23 to 9.42 m/s, respectively. For this reason, the wind speed range of 2.5 to 10 m/s was selected to analyze the behavior of the different masts.

**Figure 20:**
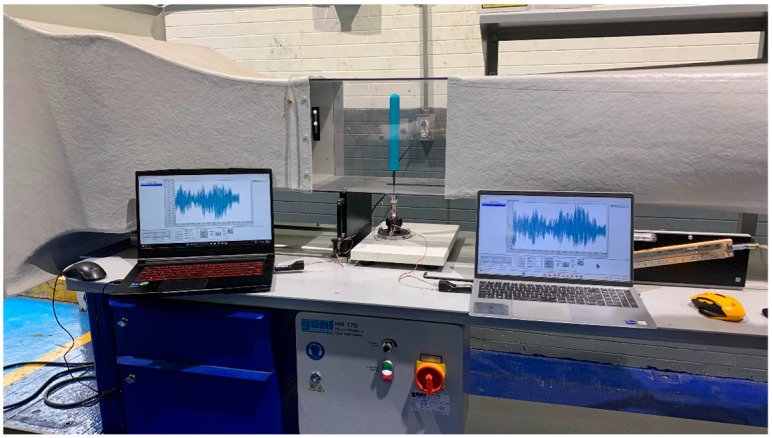
Cylindrical mast testing in the wind tunnel.

This study aims to determine whether a specific geometry generates greater lift forces than others and to quantify these differences, thereby validating the proposed hypothesis. Figure 20 shows the execution of the cylindrical mast testing.

**Figure 21:**
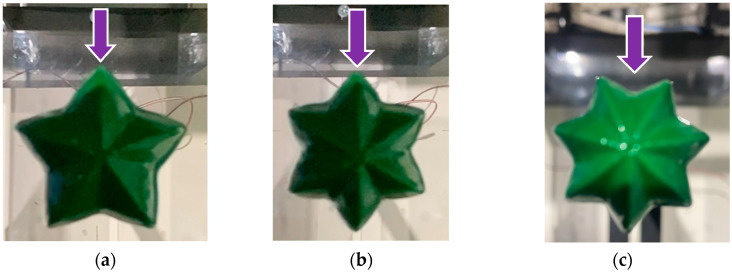
Top view of the masts angle. (**a**) 5-ribs cactus-type (0°), (**b**) 6-ribs cactus-type (0°), and (**c**) 7-ribs cactus-type (25°).

The mast angle is the angle of deviation of the mast with respect to the wind direction. An angle of 0° means that one tip of the mast rib is aligned with the wind direction (see purple lines in Figure 21a,b), and for an angle of 25°, it means that the wind direction first impacts the cavity between two adjacent ribs of the cactus (see purple line in Figure 21c).

**Figure 22:**
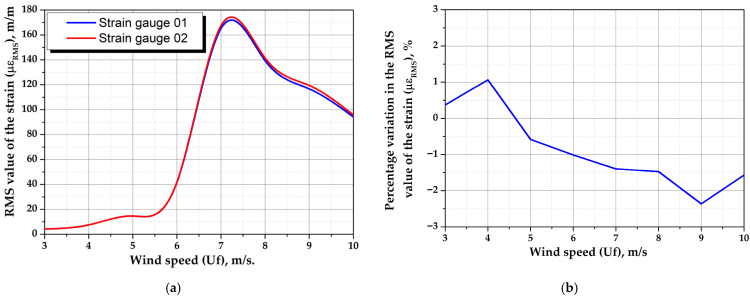
Experimental results of the 5-ribs cactus-type with a mast angle of 0°. (**a**) Measurements of both strain gauges as a function of wind speed and (**b**) percentage difference between strain gauges.

The experimental results are based on signals captured by the data acquisition system through strain gauges. These signals quantify the response of each prototype at different wind speeds, providing information on vortex-induced vibrations and the mechanical energy absorbed through oscillations. As the load cell is equipped with two strain gauges that simultaneously measure strain with opposite signs (i.e., tension and compression) resulting from the deflection of the load cell under the wind-induced force, Figure 22a shows the RMS value of the strain of both strain gauges as a function of wind speed of the 5-ribs cactus-type with a mast angle of 0°.

Figure 22b presents the percentage difference between the strain gauge measurements. As illustrated, the maximum percentage difference is approximately 2.5%, indicating that the prototype is properly aligned, meaning that one of its tips is oriented in the direction of the wind. This configuration produces a symmetrical wind-induced force, preventing twisting effects that could compromise prototype performance. Additionally, this small percentage difference indicates that the bonding process and subsequent installation of the strain gauges were performed with high precision. Based on these comparative results, the analysis will henceforth focus exclusively on the data obtained from strain gauge 1, while the data from strain gauge 2 will continue to be monitored for verification purposes.

**Figure 23:**
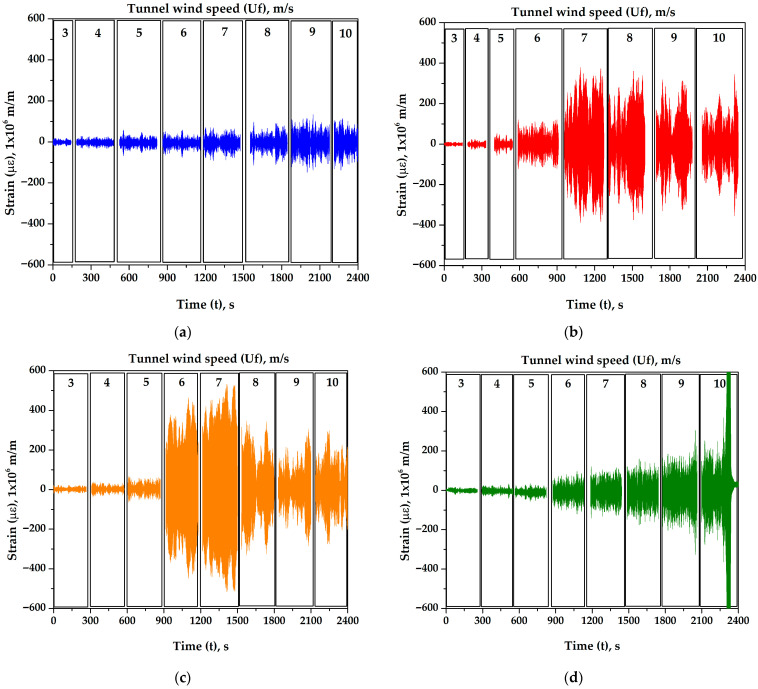
Strains of different masts as a function of tunnel wind velocity with a mast angle of 0°. (**a**) Cylindrical, (**b**) 5-ribs cactus-type, (**c**) 6-ribs cactus-type, and (**d**) 7-ribs cactus-type.

Figure 23 shows the strain response of all masts for tunnel wind velocities ranging from 3 to 10 m/s at a mast angle of 0°. The same *y*-axis scale is used to directly compare their vibratory behavior. These strain values correspond to steady-state responses; each wind speed was maintained for 200 to 300 s to record the strains, with this variation in duration explained later. Data corresponding to wind speed transitions and the stabilization period were removed to ensure accuracy. The data was cleaned to eliminate effects related to transient strain fluctuations and potential delays in vortex shedding formation caused by acceleration rates.

**Figure 24:**
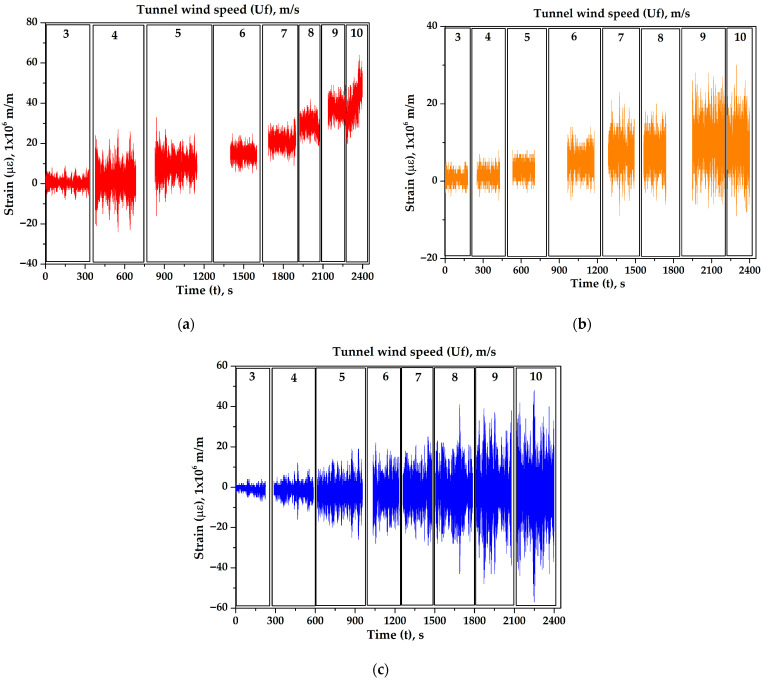
Strains of different masts as a function of tunnel wind velocity with a mast angle of 25°. (**a**) 5-ribs cactus-type, (**b**) 6-ribs cactus-type, and (**c**) 7-ribs cactus-type.

Figure 24 shows the strain of all masts for tunnel wind velocities ranging from 3 to 10 m/s at a mast angle of 25°. The figure indicates that the 25° mast angle produces lower strain values compared to the 0° angle and shifts the oscillation axis of each mast in a direction perpendicular to the wind. This occurs because the wind exerts a greater thrust force on the cactus-type masts, significantly reducing the lift force. The displacement effect of the oscillation axis is more pronounced in cactuses with fewer ribs.

As shown in Figure 23 and Figure 24, the amplitudes of oscillation for each wind speed are not constant. This behavior is due to the viscoelastic nature of the flexible rod, which on one hand stores potential energy (spring effect) and on the other hand dissipates energy during vibration (damping effect). This viscoelastic behavior is characterized by greater damping than that observed in metals. The main sources of this damping are the internal material damping caused by the polymer matrix (resin), where part of the mechanical deformation energy is converted into heat due to internal friction between polymer chains; the fiber–matrix interaction, where glass or carbon fibers embedded in the polymer matrix experience microscopic slippage or micro-fracturing at the interface, dissipating energy; and in-ternal friction, which includes micro-fibrillation and micro-cracking that produce small energy losses due to friction within the internal contacts of the composite material during vibration. Therefore, these damping forces significantly affect the amplitudes of oscillation induced by wind lift forces on the masts, leading to notable variations in their magni-tudes. However, Figure 23 shows that the maximum deformation amplitudes tend to stabilize. When this occurs, it indicates that a steady-state deformation condition has been reached. It has been observed that the maximum amplitudes are reached within a time range of 50 to 100 s, and the duration of data acquisition for each wind speed is between 200 and 300 s. The wind speed in the tunnel was increased gradually to avoid large oscillation peaks caused by abrupt changes in velocity, which could delay the establishment of steady-state conditions.

**Figure 25:**
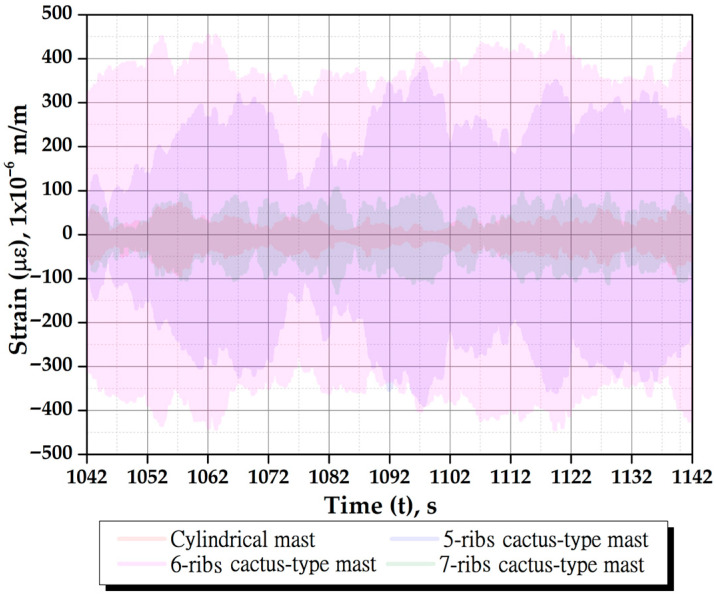
Behavior of the strains for all masts at a speed of 7 m/s with a mast angle of 0°.

**Figure 26:**
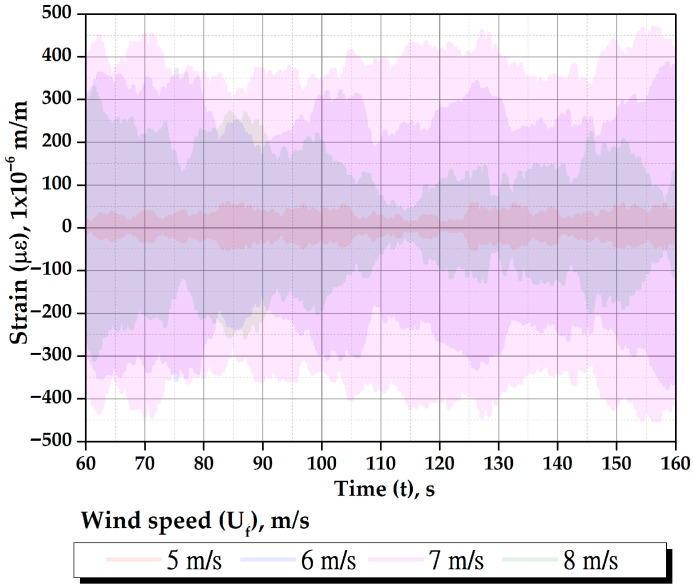
Strains of the 6-ribs cactus-type mast for different wind speeds with a mast angle of 0°.

As illustrated in Figure 25, the strains of all masts are presented for a wind speed of 7 m/s at a mast angle of 0°, which is close to the resonance wind speed. The RMS strain values (με_RMS_) are as follows: 24 με for the cylindrical mast, 165 μɛ for the 5-rib cactus-type mast, 271 μɛ for the 6-rib cactus-type mast, and 44 μɛ for the 7-rib cactus-type mast. At this wind speed, the 6-rib cactus-type mast demonstrates the best performance. As illustrated in Figure 26, the superimposed strain responses of the 6-rib cactus-type mast with a mast angle of 0° are presented as a function of varying wind speeds. For example, the RMS strain (με_RMS_) values are 21 μɛ at 5 m/s, 212 μɛ at 6 m/s, 271 μɛ at 7 m/s, and 135 μɛ at 8 m/s. The strain magnitude increases at a wind speed of 7 m/s, as the third natural frequency approaches this velocity (refer to Table 4).

## 6. Discussion

**Figure 27:**
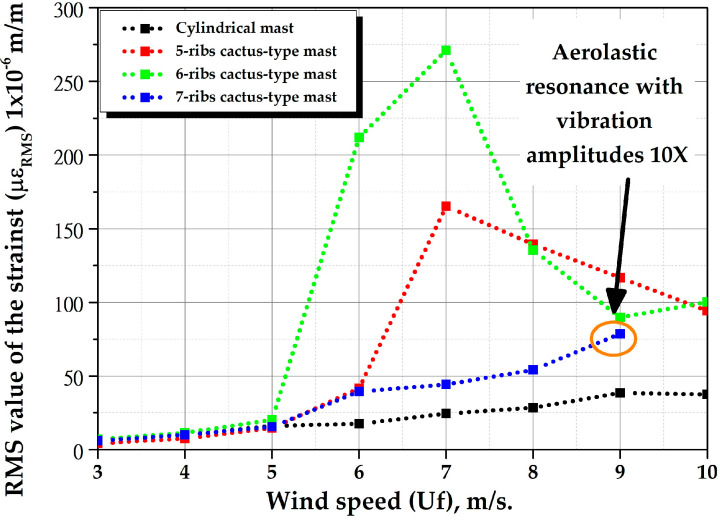
Root mean square (RMS) of the strains of all masts as a function of wind speed ($U_{f}$) with a mast angle of 0°.

Figure 27 shows the RMS value of strains (µε_RMS_) for all masts within a wind speed range of 3 to 10 m/s with a mast angle of 0°. The figure illustrates that vortex-induced vibration amplitudes are greatest near a wind speed of 7 m/s for 5 ribs- and 6-ribs cactus-type masts. This is because this speed is in the “lock-in range”, where aeroelastic resonance occurs. Similarly, the 7-ribs cactus-type mast enters resonance when crossing a speed of 9.0 m/s, strains are not shown because they reach magnitudes of up to 3000 µε.

The numerical study of natural vibration frequencies determined that resonance occurs at wind speeds of 7.23 m/s (third natural frequency) and 9.42 m/s (fourth natural frequency), both of which are within the range of 3 to 10 m/s. Therefore, there is consistency between the numerical results and the experimental data. On the other hand, this figure shows that the cylindrical mast has the best performance at wind speeds of 3 m/s. For wind speeds between 4 and 7 m/s, the 6-ribs cactus mast performs better. For wind speeds between 8 and 9 m/s, the 5-ribs cactus mast performs better. For wind speeds above 10 m/s, the 7-ribs cactus mast is the best option. This behavior is because the continuous and circular nature of the cylindrical geometry favors vortex shedding that alternately detaches from both sides of the mast on a periodic basis. Unlike an irregularly shaped object, the circular shape creates a constant, stable, and symmetrical interruption of the flow. However, at moderate and high wind speeds, cactus-like geometries promote more flow separation at the edges, creating areas of low pressure and accelerated speeds, increasing instability and the detachment of alternating turbulent vortices that improve lift. In other words, the wind flows over the ribs of the cactus cannot follow the shape of the contour and separates from the surface easily, creating vortices that detach and are carried away by the current, generating more lift.

**Figure 28:**
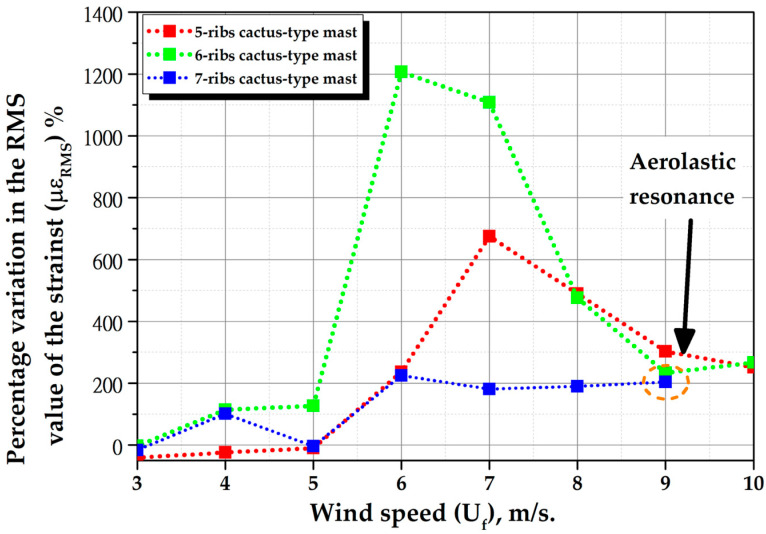
Variation in (%) of the RMS value of strains of cactuses-type masts with respect to cylindrical masts for different wind speeds with a mast angle of 0°.

**Figure 29:**
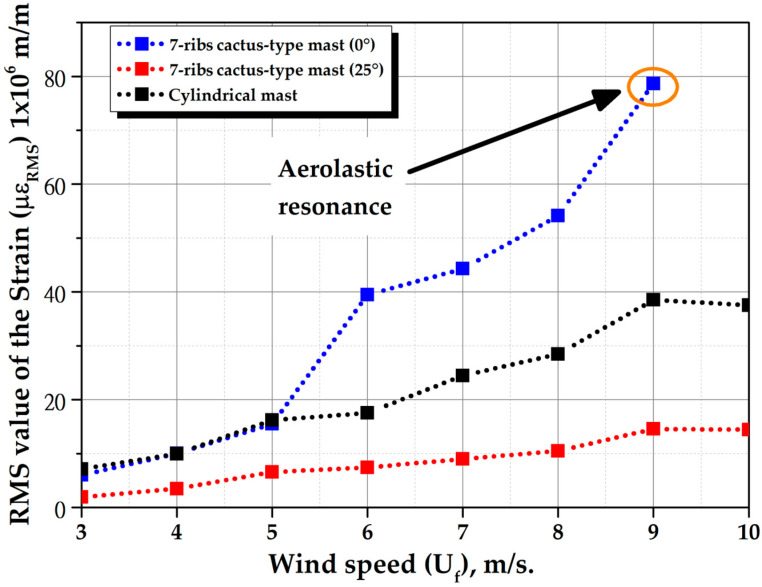
Root mean square (RMS) of the strains of 7-ribs cactus-type as a function of wind speed ($U_{f}$) with a mast angle of 0° and 25°.

Figure 28 shows the percentage variation in the RMS value of the strains (με_RMS_) of cactus-type masts with a mast angle of 0° compared to cylindrical masts for different wind speeds. For instance, when wind speeds reach 6 m per second, the 5-ribs cactus-type mast experiences a 237% increase, the 6-ribs cactus-type mast a 1207% increase, and the 7-ribs cactus-type mast a 225% increase. Figure 29 shows the RMS value of the strain as a function of time for the 7-rib cactus-type mast with a mast angle of 0° and 25°, and a comparison with the strain of a cylindrical mast. This figure shows that the angle of the mast has a significant effect on the magnitude of the strains. For a mast angle of 25°, the strain is even less than that of a cylindrical mast. Therefore, to maximize the energy capture of the bladeless wind turbine, an orientation system must be in place that allows the turbine to always align itself with the wind direction, for example, a passive orientation system consisting of a tail with a rotating base.

**Figure 30:**
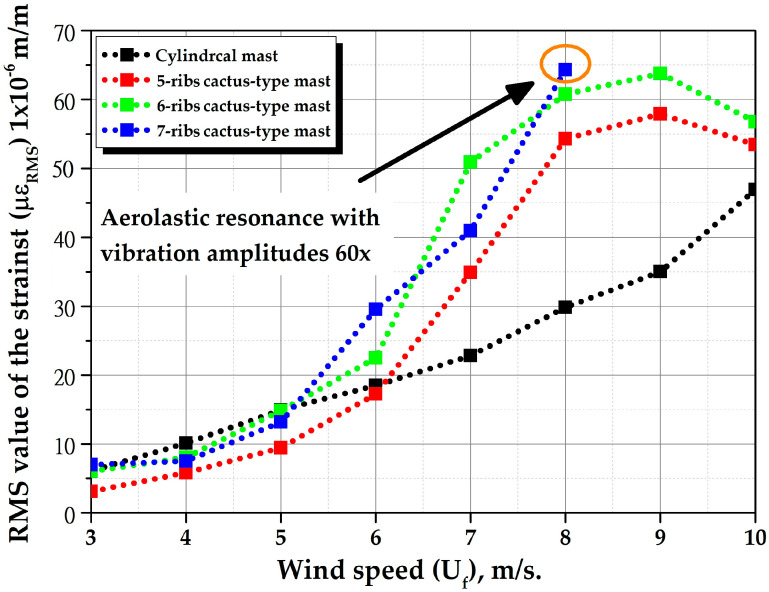
Root mean square (RMS) value of strains of all masts with flexible carbon fiber beams as a function of wind speed ($U_{f}$) with a mast angle of 0°.

To further study vortex-induced vibrations, additional tests were performed on all prototypes with a mast angle of 0°, with flexible beams made of carbon fiber reinforced polymer ($E_{1}=120\;GPa\;[Young’s\;modulus\;0{}^{\circ}],\;\rho =1560\;kg/m^{3}$), see Figure 17. The diameter and effective length of the flexible beam remained constant. Figure 30 shows the RMS value of the strains (με_RMS_) of the masts as a function of wind speed with a mast angle of 0°, ranging from 3 to 10 m per second. The figure indicates that the exhibited behavior is analogous to that of the masts with fiberglass-reinforced polymer flexible beams with a mast angle of 0°, with the exception that the amplitudes of the vortex-induced vibrations are significantly diminished, approximately four times smaller, this phenomenon can be attributed to an enhanced rigidity of flexible beams composed of carbon fiber reinforced polymer, which results in elevated natural frequencies. For instance, now the first natural frequency is 10.437 Hz.

Consequently, the wind speed at which aeroelastic resonance occurs is now approaching 9 m/s for 5 ribs and 6-ribs cactus-type masts. Conversely, the strains (με_RMS_) of the cylindrical mast and the 7-ribs cactus-type mast increase with increasing wind speed. The figure indicates that the 7-ribs cactus-type mast enters resonance when it crosses a speed of 8.0 m/s, strains are not shown since magnitudes of up to 4000 με are reached. This figure also shows that for wind speeds below 5 m/s, the cylindrical mast performs best; for wind speeds between 5 m/s and 8 m/s, the 6-ribs cactus-type mast performs best; and for wind speeds above 8 m/s, the 7-ribs cactus-type mast performs best.

## 7. Conclusions

The numerical study using CFD established the foundation for this research and supported the validation of the hypothesis that “*The cross-sectional geometry of a columnar-cactus mast type improves vortex formation when fluid flows over its surface, thereby increasing the aerodynamic performance of a bladeless wind turbine*”. The 5-rib cactus-type mast exhibited higher lift forces at all wind speeds. For example, at a wind speed of 6 m/s, the increase in lift force reached approximately 60%.

Maintaining a uniform mass among all scaled prototypes—with a maximum difference of less than 0.48% between the lightest and heaviest models—and preserving a consistent effective length of the flexible fiberglass-reinforced polymer beam during testing resulted in a uniform natural vibration frequency of 8.3 Hz across all prototypes. This ensured that the variations in vortex-induced vibrations were exclusively attributable to aerodynamic effects caused by the cactus geometries, eliminating the influence of variations in natural frequency associated with mast mass or flexible beam dimensions.

During testing, the percentage variation between the two strain gauges was less than 2.5%, confirming the symmetry of the forces induced by the wind. This prevented the prototypes from experiencing torsional effects that could diminish their aerodynamic performance. The low percentage also indicated that the prototypes were properly aligned with the wind direction—that is, one of the cactus-type mast rib tips was oriented directly into the wind—and that the bonding and installation of the strain gauges were performed correctly.

The experimental results of the bio-inspired design of the bladeless wind turbine masts confirmed the hypothesis that “The cross-sectional geometry of a columnar-cactus mast type improves vortex formation when fluid flows over its surface, thereby increasing the aerodynamic performance of a bladeless wind turbine”. This conclusion is supported by the fact that both mast configurations, with a mast angle of 0° and flexible beams made of fiberglass-reinforced polymer or carbon fiber–reinforced polymer, exhibited similar behavior. For example, the magnitude of the vortex-induced vibrations varied significantly. At a wind speed of 6 m/s, the 5-rib cactus-type mast increased by 237%, the 6-rib cactus-type mast by 1207%, and the 7-rib cactus-type mast by 225%. For cactus-type masts with flexible carbon fiber–reinforced polymer beams, at a wind speed of 8 m/s, the increases were 182%, 204%, and 216% for the 5-, 6-, and 7-rib masts, respectively.

In the design of bladeless wind turbines operating at average annual wind speeds below 5 m/s, the use of a cylindrical mast is more appropriate. For bladeless wind turbines with average annual wind speeds above 5 m/s, the use of cactus-type masts is highly recommended, particularly for speeds between 5 and 7 m/s, where the 6-rib cactus-type mast shows the best performance. For wind speeds between 8 and 9 m/s, the 5-rib cactus-type mast is recommended, while for speeds above 10 m/s, the 7-rib cactus-type mast is advised. Because the mast angle significantly reduces the magnitude of strain, implementing a yaw system that aligns the rib tips with the wind direction (0°) is essential to prevent a decrease in vortex-induced vibrations and, consequently, a reduction in power output.

## Figures and Tables

**Table 1:** Natural frequencies of mast–flexible beam–“load cell” assembly.

Frequency	Value (Hz)	Wind Speed (m/s)	Re
First	8.9	1.16	3.359
Second	13.6	1.91	5530
Third	51.62	7.23	20,934
Fourth	67.27	9.42	27,276
Fifth	111.82	15.66	45.343

**Table 2:** Air properties for CFD simulation of the bladeless wind turbine.

Property	Symbol	Value
Air temperature	T (K)	293.2
Air pressure	P (Pa)	101.325
Air density	ρair (kg/m^3^)	1.225
Dynamic viscosity of air	μ (kg/m·s)	1.1846 × 10^−5^

**Table 3:** Mass of the different scaled prototypes.

Mast	Mass (g)
Cylindrical	76.89
5-ribs cactus-type	76.99
6-ribs cactus-type	77.26
7-ribs cactus-type	77.14

**Table 4:** Natural frequencies of prototypes measured experimentally.

Mast	Frequency (Hz)
Cylindrical	8.3349
Cactus 5 ribs	8.2585
Cactus 6 ribs	8.3321
Cactus 7 ribs	8.2494

## Data Availability

Data is contained within the article.
